# Re-interventions after endovascular aortic repair for infrarenal abdominal aneurysms: a retrospective cohort study

**DOI:** 10.1186/s12872-016-0309-0

**Published:** 2016-06-06

**Authors:** Håkan Roos, Henrik Djerf, Ludvig Brisby Jeppsson, Victoria Fröjd, Tomas Axelsson, Anders Jeppsson, Mårten Falkenberg

**Affiliations:** Department of Vascular Surgery, Sahlgrenska University Hospital, SE-413 45 Gothenburg, Sweden; Department of Cardiothoracic Surgery, Sahlgrenska University Hospital, Gothenburg, Sweden; Department of Molecular and Clinical Medicine, Institute of Medicine, Sahlgrenska Academy, University of Gothenburg, Gothenburg, Sweden; Department of Radiology, Sahlgrenska University Hospital, Gothenburg, Sweden

**Keywords:** Reoperation, Endovascular technique, Aortic aneurysm, Endoleak

## Abstract

**Background:**

Early morbidity and mortality are generally lower after endovascular aortic repair (EVAR), than after open repair but re-interventions and late complications are more common. The aim of the present study was to make a detailed description of re-interventions after EVAR-including incidence, indications, procedures, and outcome-with special reference to non-access-related re-interventions.

**Methods:**

This is a retrospective single-center cohort study of re-interventions after standard EVAR with special reference to non-access-related re-interventions. Consecutive patients (*n* = 405) treated with standard EVAR for non-ruptured (*n* = 337) or ruptured (*n* = 68) infrarenal aneurysms between 2005 and 2013 were analysed. Median follow-up was 29 months (range 0–108).

**Results:**

Eighty-nine patients (22 %) underwent 113 re-interventions during follow-up. Twenty-seven patients (7 %) had 28 access related re-intervention, 65 patients (16 %) had 85 non-access related reinterventions. Non-access related re-interventions were more common in ruptured aneurysms than in unruptured aneurysms (22 vs. 15 %, *p* = 0.002). The most frequent indications were endoleak type I (*n* = 19), type II (*n* = 21), or type III (*n* = 5); stent graft migration (*n* = 9); and thrombosis (*n* = 14). The most frequent procedures were embolization of endoleak type II (*n* = 21), additional iliac stent graft (*n* = 19), proximal extension (*n* = 12), thrombolysis (*n* = 8), iliac limb bare-metal stenting (*n* = 6), and stent graft relining (*n* = 7). Endovascular technique was used in 83 % of re-interventions.

Thirty-day mortality after non-access-related re-interventions was 15 % when initiated from symptoms (rupture or infection) and 0 % when initiated from follow-up findings (*p* = 0.014). Cumulative survival five years after EVAR was 72 % in patients with a re-intervention and 59 % in patients without (*p* = 0.21).

**Conclusions:**

Non-access-related re-intervention rates are still considerable after EVAR and more frequent after ruptured aneurysms. Endoleak embolization is the most frequent procedure, followed by additional iliac stent grafts. Outcomes after re-interventions are generally good, except when initiated by rupture or infection.

## Background

Endovascular aortic repair (EVAR) was first reported by Volodos in 1988 [1] and by Parodi in 1991 [2]. During the last decade, an increasing number of abdominal aortic aneurysms (AAAs) and iliac aneurysms have been treated with EVAR rather than with open repair. In the Swedish National Registry (Swedvasc), 59 % of all aortic repair procedures were performed with endovascular techniques in 2013, as compared to 25 % ten years earlier [3, 4]. EVAR has been compared with open repair for the treatment of AAA in several randomized and non-randomized studies. Early morbidity and mortality are generally lower after EVAR, but re-interventions and late complications appear to be more common [5–12], although there have been contradictory reports [13]. While the results of the primary EVAR procedure are well-described in the literature [5–7], there have been few publications reporting the whole spectrum of re-interventions after EVAR, including those related to the arterial access. The aim of the present study was therefore to make a detailed description of re-interventions after EVAR-including incidence, indications, procedures, and outcome-with special reference to non-access-related re-interventions.

## Methods

### Patients

**Fig. 1:**
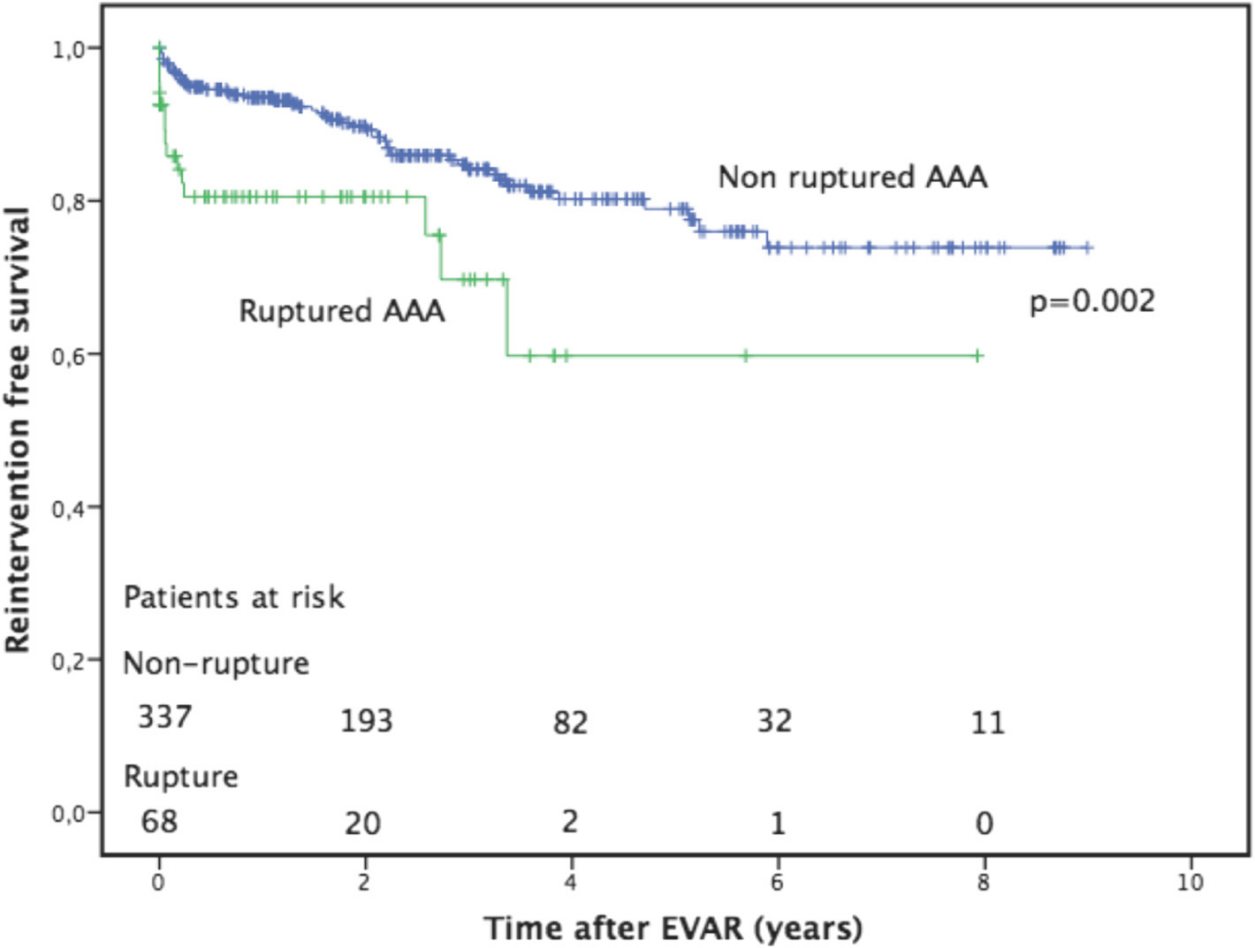
Freedom from non-access related re-intervention presented in months after primary EVAR, comparing patients treated for rupture vs non-ruptured aneurysms. Cumulative re-intervention free survival two years after primary repair was 80 % for patients treated for rupture and 89 % for patients treated for non-ruptured AAA. A standard error of 10 % was reached after 3.4 years in the patient with ruptured aneurysms and after >9 years in the non-ruptured patients

A consecutive series of patients treated with EVAR for abdominal or iliac aneurysms at Sahlgrenska University Hospital between 2005 and 2013 were retrospectively reviewed. During the 9-year period, 405 patients (mean age 75 ± 7 years, 84 % men) were treated for unruptured (*n* = 337) or ruptured (*n* = 68) infrarenal aneurysms. Aneurysm pathology was atherosclerotic in 379 cases, inflammatory in 12 cases, mycotic in 13 cases and aortic dissection with common iliac artery aneurysm in 1 case. Median follow-up time was 29 months (range 0–108). One patient was lost to follow up. This patient was a foreign citizen treated for a ruptured AAA and discharged at day 11 postoperatively. There were no re-interventions or complications during hospital stay in this patient. Patient characteristics are presented in Table 1 and Fig. 1. The rate of endovascular treatment of AAA at our institution was 28 % in 2005 and 56 % in 2013.

**Table 1:** Patients characteristics with comparison for groups with and without non-access related re-interventions after EVAR

	All	No re-interventions	Re-interventions	*P* value
	*n* = 405	*n* = 340	*n* = 65	
Age	74.8 (7.3)*	74.9 (7.4)*	74.2 (7.0)*	0.87
Male Gender	338 (83.5 %)	278 (81.8 %)	60 (92.3 %)	0.046
Body mass index	27.1 (5.2)* (*n* = 225)	27.1 (5.2)* (*n* = 195)	27.6 (4.8)* (*n* = 30)	0.76
Type of aneurysm				
Atherosclerotic	379 (94 %)	319 (94 %)	59 (91 %)	0.51
Inflammatory	12 (3.0 %)	9 (2.6 %)	3 (4.6 %)2	0.27
Mycotic	13 (3.2 %)	11 (3.2 %)	(3.1 %)	0.94
Other	1 (0.2 %)	1 (0.5 %)	1 (1.5 %)	0.28
Ruptured aneurysm	68 (16.8 %)	53 (15.6 %)	15 (23.1 %)	0.002
AAA diam (mm)	66 (13.0)* (*n* = 381)	65 (12.9)* (*n* = 322)	68 (13.2)* (*n* = 59)	0.026
CIA diam (mm)	45 (13.0)* (*n* = 49)	45 (13.8)* (*n* = 39)	47 (12.0)* (*n* = 10)	0.52
Serum creatinine (umol/L)	104 (66.7)*	103 (60)*	111 (94.3)*	0.082
Diabetes mellitus	69 (17.0 %)	60 (17.6 %)	9 (13.8 %)	0.65
Known pulmonary disease	89 (22.0 %)	77 (22.6 %)	12 (18.5 %)	0.68
Previous cardiac disease**	189 (46.7 %)	160 (47.1 %)	29 (44.6 %)	0.54
Dialysis	13 (3.2 %)	10 (3.0 %)	3 (4.6 %)	0.27
Previous cerebral infarction/TIA	51 (12.6 %)	46 (13.6 %)	5 (7.7 %)	0.22
Hypertension	295 (72.8 %)	253 (74.4 %)	42 (64.6 %)	0.33
Smoking (ever)	267/340 (78.5 %)	232/290 (80.0 %)	35/50 (70.0 %)	0.27

Key: *AAA* abdominal aortic aneurysm, *CIA* common iliac artery, *TIA* transient ischemic attack

*Presented as mean and SD

**Previous cardiac surgery, myocardial infarction or disabling angina pectoris

### Study protocol

Patient records, institutional databases, and national registry data were reviewed regarding primary repair, complications, re-interventions, and survival. National registry data were validated using the medical records. Juxta- and suprarenal aneurysms treated with fenestrated, branched, or chimney stent grafts during the period were excluded. Rupture of an aneurysm was defined as retroperitoneal hematoma and/or extravasation of contrast on preoperative computed tomography (CT).

A re-intervention was defined as any procedure (open or endovascular) where the decision to re-intervene was taken after the patient had left the operating theatre after the EVAR procedure. Re-interventions were categorized as either being access-related or not access-related. Access-related re-interventions included suture of access bleeds, distal thrombembolectomy, patch angioplasty, or thromboendarterectomy of the common femoral artery. Non-access-related re-interventions included all remaining secondary procedures related to the primary EVAR. The numbers and types of re-interventions were recorded, as well as the timing in relation to the primary EVAR. The follow-up program after EVAR consisted of CT investigations one and 12 months postoperatively, and annually thereafter. Indications for treatment were: endoleak type I and III was treated in the absence of contraindications and endoleak type II was treated only when concurrent aneurysm sac expansion > 5 mm was observed. Migration without endoleak was treated if the proximal or distal sealing zones became clearly shorter than specified in the instructions for use for the respective stent graft. Early mortality was defined as death within 30 days or in hospital. Mortality data were collected from the Swedish Civil Registry. Re-interventions were reported according to the standards for endovascular aortic aneurysm repair published by the Society for Vascular Surgery [14].

### Stent grafts

The following types of stent grafts were used: Endurant® (Medtronic, Santa Rosa, CA, USA) (*n* = 237, 59 %), Zenith Flex® (Cook Inc., Bloomington, IN, USA) (*n* = 86, 21 %), Excluder® (W. L. Gore, Flagstaff, AZ, USA) (*n* = 55, 14 %), Talent® (Medtronic) (*n* = 15, 4 %), Zenith LP® (Cook Inc.) (*n* = 8, 2 %), and Ovation® (Trivascular, Santa Rosa, CA, USA) (*n* = 2, 0.5 %). Fifteen Zenith iliac-branched devices were used. Two patients were converted to open surgery and therefore did not have a stent graft in place at the end of surgery. In the first patient access problems made it impossible to introduce a stent graft, the second patient had a stent graft mistakenly covering coeliac trunk branches with low origins.

### Statistical analysis

Continuous data are presented as mean and standard deviation (for data with normal distribution) or median and interquartile range (for data that were not normally distributed). Normality of data was checked with the Kolmogorov-Smirnov test. Categorical data are presented with numbers and percentage and they were compared between groups with Fisher’s exact test. Cox regression was used to identify independent predictors for non-access-related re-interventions. Kaplan-Meier curves were used to analyze cumulative long-term survival, followed by log-rank test for group comparisons. Any p-value < 0.05 was considered to be statistically significant. All statistical calculations were performed with SPSS 22 (IBM Corp., Armonk, NY, USA).

## Results

### General

Overall 30-day mortality after the primary EVAR procedure was 2.5 % (10/405) - 0.6 % (2/337) for unruptured aneurysms and 12 % (8/68) for ruptured aneurysms (*p* < 0.001). Early mortality in the non-ruptured group was caused by a stent graft inadvertently placed covering the renal arteries resulting in uremia in one patient and lower extremity ischemia and multi organ failure after conversion to open surgery in one patient. The mortality in the rupture group was in all cases caused by complications related to massive bleeding and multi organ failure. Five-year survival was 62 % in the whole material, 64 % in unruptured aneurysms and 39 % in ruptured aneurysms, respectively (*p* = 0.002).

Eighty-nine of the 405 patients (22 %) underwent 113 re-interventions during the follow-up period. Twenty-eight re-intervention episodes in 27 patients (7 %) were related to the arterial access in the groins. Eighty-five re-intervention episodes in 65 patients (16 %) were not access-related. Three patients underwent both access- and non-access-related re-interventions. Median hospital stay after a non-access-related re-intervention was two days (range 0–46).

### Access-related re-interventions

Twenty-eight access-related re-interventions were recorded in our series. Most were done in the early postoperative phase: 20/28 during the same hospital stay, 0–2 days after EVAR. The prevalence of access-related re-interventions was not significantly different for ruptured and unruptured aneurysms: 8.8 % vs. 6.8 % (*p* = 0.60). 740 large access punctures were performed in 405 patients. Standard techniques for arterial closure was either the percutaneous pre-suture technique using Prostar XL® (Chicago, IL, USA) or fascia suture as described by Larzon et al. [15]. Surgical cut down was used only in selected patients with heavy calcifications in the common femoral artery. Re-interventions were performed in 13/438 (3 %) groins after closure with Prostar XL, 7/228 (3 %) after fascia suture, 6/42 (14 %) after cut down, and in 2/16 cases (32 groins) (12 %) after femoro-femoral bypass, (*p* = 0.009 for cut down vs fascia suture and Prostar XL). Indications for access-related re-interventions were bleeding (*n* = 11), thrombosis (*n* = 12), pseudoaneurysm (*n* = 4), stenosis (*n* = 1). Access-related re-intervention procedures were suture of bleedings (*n* = 11), distal embolectomies (*n* = 11), patch angioplasties (*n* = 5), and thromboendarterectomy of the common femoral artery (*n* = 1). There were two early deaths in patients with access-related re-interventions, both of whom were treated for rupture at primary EVAR. Both of these re-interventions were done on day 1 after EVAR, and the patients died on days 3 and 7.

### Non-access-related re-interventions

#### Incidence

**Fig. 2:**
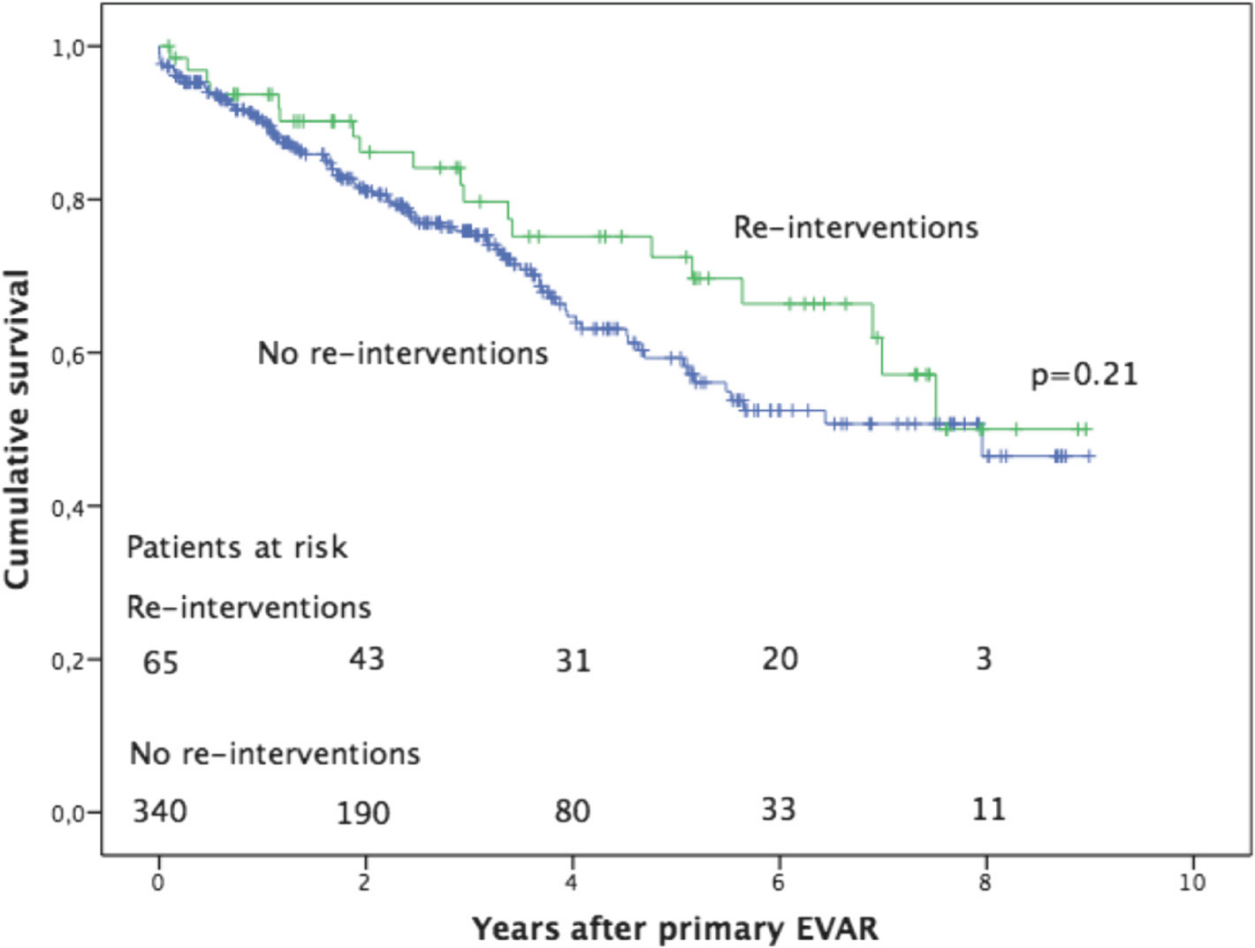
Survival after EVAR comparing patients with and without re-interventions. Cumulative survival at 5 years was 72 % for patients with re-interventions and 59 % for patients without re-interventions. A standard error of 10 % was reached after 7.5 years in the patient with re-interventions and after >9 years in patients without re-interventions

**Fig. 3:**
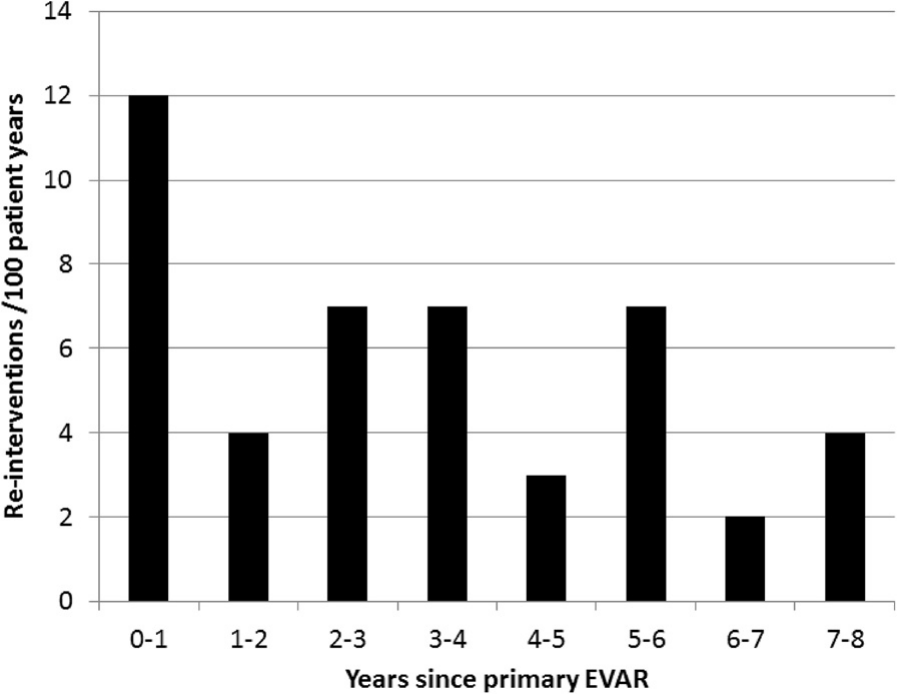
Annual incidence of non-access-related re-interventions after primary EVAR

The median time between EVAR and the first non-access-related re-intervention was 14 months (range 0–91) (Fig. 2). Eleven patients (3 %) had two non-access-related re-interventions, 4 patients (1 %) had three, and one patient (0.2 %) had four. The overall incidence of non-access-related re-intervention was 7 per 100 patient years with the highest incidence (12/100 patient years) during the first year after primary EVAR (Fig. 3). The number of patients with re-interventions were 60/379 (16 %) for atherosclerotic aneurysms, 3/12 (25 %) for inflammatory aneurysms, 2/13 (15 %) for mycotic aneurysms and 0/1 (0 %) for others (iliac aneurysm in previous dissection).

#### Indications

Fifty-one (60 %) of the non-access-related re-interventions were initiated from findings on follow-up imaging and 34 (40 %) from symptoms. Presenting symptoms were new onset of leg pain or claudication caused by iliac limb thrombosis (*n* = 12) or stenosis (*n* = 1), fever, abdominal pain or sepsis caused by graft infection (*n* = 7 in 5 patients), abdominal pain and shock due to rupture (*n* = 4), abdominal compartment syndrome after rupture at primary repair (*n* = 3), abdominal pain due to bowel ischemia (*n* = 2), abdominal or back pain caused by aneurysm expansion (*n* = 2), continued bleeding after RAA (*n* = 2) and notable aneurysm expansion by patient (*n* = 1). The most common indications for non-access-related re-intervention were endoleak type I (*n* = 19), type II (*n* = 21), or type III (*n* = 5); stent graft migration (*n* = 9); and graft limb thrombosis (*n* = 14 in eleven patients). Six of the patients with thrombosis had an iliac landing zone in the external iliac artery. The frequency of graft limb thrombosis was 6.1 % if the landing zone was the external iliac artery compared to 0.7 % if the landing zone was in the common iliac artery (*p* < 0.001). Aneurysm expansion was present in 39 % of the non-access-related re-interventions (Fig. 3 and Table 2). Four patients had secondary rupture after EVAR as the indication for re-intervention; details are given in Table 2.

**Table 2:** Rupture as indication for re-intervention. Details of primary EVAR, outcome and follow up

	Primary EVAR	Time to reinterven-tion (days)	Re-intervention	Survival	Follow up (days)
Patient 1	Non-rupture	84	Proximal cuff	Dead	16
	Bifurcated		Infected EVAR –Chimney graft to renal arteries		
Patient 2	Non-rupture	166	Proximal	Dead	3
	Bifurcated		Extension- Cuff		
Patient 3	Rupture	2	Bifurcated EVAR in previous tube graft	Alive	276
	Tube graft				
Patient 4	Non-rupture	1875	Distal extension	Alive	32
	Bifurcated				

Separate analysis of the time to re-intervention was done for all types of re-interventions with >5 procedures for each group. Embolization of endoleaks and additional iliac stent grafts were performed significantly later after primary EVAR compared to thrombolysis and distal bare metal stent (*p* = 0.05) and there was also a tendency in the same direction between embolization and additional iliac stent graft compared to proximal extensions (*p* = 0.06), (Table 3).

**Table 3:** Number of re-intervention procedures performed for each group of re-interventions. Number of patients, days after primary repair and number of re-interventions performed due to findings on follow up imaging or due to symptom

		Number of reintervention procedures	Number of patients	Days after primary EVAR^a^	Detected by	
					Follow up	Symptoms
ENDOVASCULAR	Embolisation	21	17	1029 (578–1357)^a^	20	1
	Additional iliac stent graft	19	17	821 (539–1414)^a^	15	4
	Proximal extension	12	12	239(68–1163)^a^	6	6
	Thrombolysis	8	7	41 (18–90)^a^	0	8
	Iliac bare metal stent	6	6	27 (8–242)^a^	3	3
	Relining	5	5	0; 40; 44; 415; 2151^b^	4	1
	Drainage of aneurysm sac in infected aneurysm	1	1	773^b^	0	1
	Bifurcated stent graft in previous isolated iliac stent graft/aortic tube graft	2	2	2; 7^b^	1	1
	Renal artery stent	1	1	581^b^	1	0
	Palmaz stent	1	1	105^b^	1	0
	Balloon dilatation of iliac limb stent graft	1	1	77^b^	0	1
	Stent graft external to internal iliac artery in combination with femoro-femoral bypass	1	1	13^b^	1	0
	Onyx in proximal sealing zone due to endoleak type I	1	1	202^b^	1	0
OPEN SURGERY	Open surgery with stent graft extirpation^c^	4	4	641; 785; 1069; 1420^b^	2	2
	Femoro-femoral crossover bypass	4	4	0; 13; 20; 80^b^	1	3
	Laparotomy due to abdominal compartment syndrome	3	3	0; 1; 20^b^	0	3
	Bowel resection	2	2	5; 32^b^	0	2
	Rafi of small intestine in patient with aortoenteric fistula. (Two operations on one patient with mycotic AAA and gastrointestinal fistula)	2	2	19; 20^b^	0	2
	Laparotomy with lumbar artery ligature	1	1	2160^b^	1	0

^a^Days after primary EVAR presented as median and interquartile range

^b^Presented as days after primary EVAR for each procedure

^c^Open surgery with stent graft extirpation was performed with axillobifemoral reconstruction in three cases and abdominal tube graft in one case

#### Procedures

The non-access-related re-interventions were endovascular, in 69 of 85 cases (81 %), open in 15/85 (18 %), and a hybrid procedure with both endovascular and open technique in one case (1 %). Since a combination of different procedures was performed at the same re-intervention episode in nine patients (in eight cases two different procedures and in one case a combination of three different procedures) the total number of re-intervention procedures was 95 (Table 3).

#### Embolizations

The most frequent non-access-related re-intervention procedure was embolization of endoleaks (21 procedures in 17 patients). One patient underwent three embolization episodes and two patients two episodes. Embolization was performed with coils in 8 procedures, with both coils and a transcatheter liquid embolization agent (ethylene vinyl alcohol copolymer, Onyx®, Covidien, Mansfield, MA, USA) in 4 procedures, and with Onyx® alone in 9 procedures. Indications were persistent type-II endoleak with sac expansion in 18 procedures. Reasons for re-intervention in the three patients without sac expansion were postoperative retroperitoneal bleeding from the aneurysm fed by type-II endoleak in one patient with secondary infected aneurysm, type II endoleak with massive outflow through an aorto-caval fistula in one patient, and treatment of type II endoleak without sac expansion in one patient with preoperative suspicion of type I endoleak. Embolization procedures were done through arterial puncture in the groin, except in one case with direct percutaneous sac puncture. Target vessels were lumbar arteries in 11 procedures, inferior mesenteric artery in three, both lumbar arteries and inferior mesenteric artery in five, and the aneurysm sac in two cases.

#### Additional iliac stent grafting

Placement of an additional iliac stent graft was the second most common non-access-related re-intervention procedure (*n* = 19). In most cases, the additional stent graft extended the distal seal further down into the iliac vessels (*n* = 16) and in three cases it secured the overlap between the main bifurcated graft and the initial iliac stent graft. The additional iliac stent grafts extending the distal seal landed in the common iliac artery in 9 patients and in the external iliac artery in 7 patients. Indications for additional iliac stent grafts were insufficient distal sealing length (*n* = 10), visible endoleaks of type Ib (*n* = 7), or visible endoleaks of type III (*n* = 3). Fourteen of the 19 re-interventions were done more than two years after the primary procedure, and in one patient a distal extension sealed an aneurysm rupture 5 years after EVAR.

#### Proximal extension

Proximal cuff extension was performed in 12 patients during follow-up. Seven of these were treated during the first year of follow-up for endoleak of type 1a. Three of five patients requiring proximal extension more than one year after the index procedure were initially treated with stent grafts without anchoring barbs.

#### Stent graft relining

Stent graft relining was done in five patients, with a different indication for each one. The indications were: type-IV endoleak, limb occlusion, conversion to aortouniiliac graft due to endoleak type I, graft stenosis, and damage to the stent graft at open operation in a patient with mycotic AAA.

#### Open surgery

Three axillobifemoral bypasses with removal of an infected stent graft were done 21, 26, and 35 months after EVAR. Two were secondary infections and one was a primary mycotic AAA, diagnosed before treatment. One patient with secondary infection died 6 days after surgery and the other two were alive after 5 and 7 years of follow-up. Four femoro-femoral bypasses were done 0, 13, 20, and 80 days after EVAR. Three were due to thrombosis of an iliac limb and one was an adjunct procedure to exclude an endoleak of type Ib with an aorto-uni-iliac converter. A late conversion to open repair (after 4 years) and an open lumbar artery ligation (after 6 years) were done in two patients for persistent type-II endoleaks with aneurysm expansion. Both patients were alive after another 2 and 4 years of follow-up. Two patients had bowel resection due to post-EVAR mesenteric ischemia. At the primary procedure both patients had bilateral occlusion of the internal iliac arteries. One of the patients was operated with an iliac-branched graft to the internal iliac artery at primary repair but the graft occluded day 2 after surgery. The second patient had staged embolization of the internal iliac artery 25 days before primary repair.

### Factors associated with non-access-related re-interventions

In univariate testing, male gender (*p* = 0.046), aneurysm size at primary repair (*p* = 0.026), and ruptured aneurysm at primary repair (*p* = 0.002) were associated with non-access-related re-interventions (Table 1). In the multivariate model, rupture (hazard ratio (HR) = 2.23, 95 % confidence interval (CI) 1.13–4.40; *p* = 0.020) and male gender (HR = 2.97, CI 1.07–8.20; *p* = 0.036) remained statistically significant.

### Outcome

Thirty-day mortality after non-access-related re-interventions was 6.2 % (4/65 patients). There were four early deaths, all occurred in patients with re-interventions due to symptoms, 4/27 (15 %) and none in patients with re-intervention due to surveillance imaging 0/38 (*p* = 0.014). Cumulative survival at five years was 72 % in patients with re-intervention and 59 % in patients without re-intervention (*p* = 0.21) (Fig. 2). Survival was not significantly different between different aneurysm pathologies (*p* = 0.37) or types of stent grafts (*p* = 0.86). Cumulative re-intervention-free survival at two years was 81 % in patients treated for rupture and 90 % in patients treated for unruptured aneurysms (*p* = 0.002) (Fig. 1).

## Discussion

The main finding of the present study was that non-access-related re-intervention rates remain considerable after EVAR. Outcomes after re-interventions were generally good, except when they were initiated by rupture or infection.

Re-interventions and late complications are the Achilles heel of EVAR. While there is a relative consensus on indications for re-intervention, little is reported on their incidence and actual clinical outcomes. Stent graft development in recent years has focused on lower-profile and easier deployment rather than on increased long-term durability. One of the main challenges in EVAR development are the endoleak-related re-interventions performed to maintain the integrity of the stent graft. Not only reporting re-intervention rates but also achieving a better understanding of the various entities of re-interventions and late complications after EVAR may improve future stent graft designs and clinical follow-up programs. In this study, we followed a cohort of 405 consecutive patients treated with standard EVAR and analyzed all access- and non-access-related re-interventions.

### Access related re-interventions

The access related re-interventions came early after primary repair and had little or no influence on the long-term results. The increased incidence of re-interventions after access closure with open cut down was most likely due to the patient selection since open cut down was used only in patients with heavy calcification of the femoral arteries.

### Non-access related re-interventions

The main focus of this report is on non-access related re-interventions and further analysis is hereafter focused on this subject. Some of our results were as expected. The total prevalence of 16 % and the rate of 7 % per 100 patient years are in line with most other reports [16–19]. Most re-interventions were performed with endovascular technique, and were initiated by follow-up imaging. Indications were dominated by type-I and type-II endoleaks. Hospital stay was short for these patients, morbidity low, and survival good. Furthermore, we found that patients treated for rupture had a higher re-intervention rate than patients treated for intact AAA. As expected, there was a more dismal prognosis when re-intervention was caused by symptoms. There were four secondary ruptures (4/405, 1 %), both the incidence and the fact that the majority of these occurred late after primary repair also corresponds well with previous reports [20]. Furthermore, our findings that stent graft limb occlusions occurred early after primary repair and that most re-interventions can be performed with endovascular technique are consistent with previous reports [16].

Some of our findings were more difficult to predict. Women had significantly fewer re-interventions then men (7 vs. 18 %). Possible explanations include smaller sizes of aneurysms, necks and iliac arteries in women, but also one may also speculate that a more restrictive attitude to reinterventions in women among patients and physicians may play a role. This has not been observed earlier [16, 21] and may well be an effect of insufficient statistical power, due to the limited number of women in our series. Previous reports have shown reduced overall survival in women [16, 22], which we could not confirm although there was a trend in the same direction. Cumulative survival at five years was 49 % for women and 65 % for men (*p* = 0.18).

Median time to the first non-access-related re-intervention was relatively long: 14 months. This finding has possible clinical implications, since follow-up schemes are usually most intense during the first postoperative year. Interestingly, certain re-intervention procedures were more frequent early in the follow-up period and others later. For instance, 7 of 12 proximal extensions were done during the first postoperative year while 14 of 19 additional iliac stent grafts were placed more than two years after the index procedure. Placement of an additional iliac stent graft was the second most common re-intervention procedure. The high frequency and tardy occurrence of this re-intervention suggests that it may in fact be a more important cause of late EVAR failure than has hitherto been recognized. Indeed, one of two late ruptures in our cohort was caused by distal stent graft migration. The importance of iliac seal for long-term stability after EVAR has also been pointed out by Ohrlander et al. They found that a greater diameter of the common iliac arteries was associated with an increased re-intervention rate [23]. It has also been shown that iliac diameter increases over time in patients treated with EVAR [24]. In an experimental study, our group showed that increased iliac angulation is a determining factor in the genesis of extraction forces induced on iliac limb stent grafts [25]. An increased focus on iliac landing zone and fixation may help to improve long-term durability after EVAR.

We discerned some trends that were not statistically significant in our series, including a tendency of improved 5-year survival in patients requiring a re-intervention compared to those who did not. This trend contrasts with a recent multicenter series from 17 centres, which did find a significant increase in aneurysm-related mortality in patients who had undergone re-interventions [16]. However, the finding in our series was based on all-cause mortality, not aneurysm-related mortality, which may at least partly explain the difference [16].

The present study had both strengths and limitations. The limitations included the ones that are inherent in a retrospective observational study, e.g., selection bias and the importance of non-registered confounders. Furthermore median follow-up was only 29 months. Another limitation was the single-centre design, as the patient numbers may have been insufficient for detection and validation of more rare events. A single-centre design also has certain advantages, since it allows registration of more detailed information. Another obvious strength was the almost complete follow-up.

## Conclusions

Non-access-related re-interventions rates after infrarenal EVAR is still considerable and more so when primary EVAR was performed due to ruptured aneurysms. Endoleak embolizations and additional iliac limb stent grafts were the most common and performed later after primary repair compared to other re-interventions. Most were done during short-stay admissions with minimally invasive techniques, and prognosis was generally good, except when the re-intervention was caused by aneurysm rupture or stent graft infection.

## Abbreviations

AAA, abdominal aortic aneurysm; CI, confidence interval; CIA, common iliac artery; EVAR, endovascular aortic repair; RAA, ruptured aortic aneurysm; TIA, transient ischemic attack
